# A Seasonal Variation of Clinical and Neurological Outcomes in Patients with Out-of-Hospital Cardiac Arrest Treated with Extracorporeal Cardiopulmonary Resuscitation: A Secondary Data Analysis of the SaveJ II Study

**DOI:** 10.3390/jpm14030306

**Published:** 2024-03-14

**Authors:** Kei Ito, Wataru Takayama, Yasuhiro Otomo, Akihiko Inoue, Toru Hifumi, Tetsuya Sakamoto, Yasuhiro Kuroda, on behalf of the SAVE-J II Study Group

**Affiliations:** 1 Trauma and Acute Critical Care Center, Tokyo Medical and Dental University Hospital of Medicine, 1-5-45 Yushima, Bunkyo-ku, Tokyo 113-8510, Japan; tak2accm@tmd.ac.jp (W.T.); otomo.accm@tmd.ac.jp (Y.O.); 2 Department of Emergency and Critical Care Medicine, Hyogo Emergency Medical Center, Kobe 651-0073, Japan; a-inoue@hemc.jp; 3 Department of Emergency and Critical Care Medicine, St. Luke’s International Hospital, Tokyo 104-8560, Japan; hitoru@luke.ac.jp; 4 Department of Emergency Medicine, Teikyo University School of Medicine, Tokyo 173-8606, Japan; sakamoto.tetsuya@nifty.ne.jp; 5 Department of Emergency Medicine, Kagawa University School of Medicine, Takamatsu 761-0793, Japan; kuroday@med.kagawa-u.ac.jp

**Keywords:** extracorporeal cardiopulmonary resuscitation, ECPR, out-of-hospital cardiac arrest, seasonal variation

## Abstract

The prognosis for patients with out-of-hospital cardiac arrest (OHCA) has been reported to be worse in the cold season. On the other hand, it is unclear whether a similar trend exists in OHCA patients who are treated with extracorporeal cardiopulmonary resuscitation (ECPR). This study was a retrospective multicenter registry study. We examined the association between ECPR and season. We compared the prognosis in four seasonal groups according to the day of occurrence. Multivariable logistic regression analysis was performed for the assessment of clinical and neurological outcomes. A total of 2024 patients with OHCA who received ECRP were included. There were no significant differences in in-hospital mortality (*p* = 0.649) and in the rate of favorable neurological outcome (*p* = 0.144). In the multivariable logistic regression, the seasonal factor was not significantly associated with worse in-hospital mortality (*p* = 0.855) and favorable neurological outcomes (*p* = 0.807). In this study, there was no seasonal variation in OHCA patients with ECPR.

## 1. Introduction

Out-of-hospital cardiac arrest (OHCA) remains one of the conditions with the poorest prognosis worldwide, and the survival rate after OHCA is low [1]. Extracorporeal cardiopulmonary resuscitation (ECPR) is used as a rescue therapy to improve the prognosis of patients with OHCA who do not achieve return of spontaneous circulation (ROSC), and the number of patients with OHCA treated with ECPR is increasing worldwide [2].

It has been reported that the clinical outcomes of patients with OHCA occurring during the cold season are worse than those in the hot season [3,4,5]. Specific diseases (e.g., myocardial infarction, respiratory diseases, and ischemic heart diseases) [6] that often occur and are complicated by cold conditions may explain the lower survival rates of patients with OHCA in the cold season. However, few reports have examined the seasonal differences in the characteristics and outcomes of OHCA patients treated with ECPR [3,4,5].

ECPR can provide hemodynamic stability, and appropriate target temperature management (TTM), leading to a higher survival rate in patients with OHCA than those without ECPR [7,8]. Patients with OHCA in cold situations can avoid hyperthermia, which could positively impact their neurological outcomes. Therefore, it is unclear whether the administration of ECPR could be beneficial for OHCA patients who transfer in the cold season.

Japan, a country with a humid subtropical climate, characterized by wide temperature differences between hot and cold seasons, is suitable for evaluating the seasonal differences. Therefore, in this study, we aimed to assess seasonal differences in the clinical and neurological outcomes of patients with OHCA treated with ECPR, assuming that the outcomes in the cold season are worse than those in the hot season.

## 2. Materials and Methods

### 2.1. Study Design and Setting

We analyzed data from the Study of Advanced Life Support for Ventricular Fibrillation with Extracorporeal Circulation in Japan (SAVE-J II study) [9].

This retrospective multicenter registry study examined the effectiveness of ECPR on clinical outcomes in 36 participating institutions in Japan between 1 January 2013 and 31 December 2018. This study complied with the principles of the 1964 Declaration of Helsinki and its amendments and was approved by the institutional review board of the University Hospital Medical Information Network (UMIN) Clinical Trial Registry (UMIN000036490) and Tokyo Medical and Dental University (M2019-018). The requirement for patient consent was waived by all participating institutions because of the retrospective nature of this study.

### 2.2. Study Population

The SAVE-J II study included consecutive patients aged ≥ 18 years admitted to the emergency department with OHCA and treated with ECPR. We excluded patients who received ECPR after intensive care unit admission, those with missing data on the day of admission, and those with trauma, suffocation, drowning, toxicity, accidental hypothermia, and other exogenous death arising from cardiac arrest.

### 2.3. Data Collection

The following patient data were collected from SAVE-J II: date, age, sex, body temperature, incidence of witnessed cardiac arrest, incidence of bystander cardiopulmonary resuscitation (CPR), incidence of using automated external defibrillator (AED), shockable rhythm status, cardiac rhythm before ECPR, defibrillation, ROSC before hospital arrival, low-flow time, cardiac arrest location, cause of cardiac arrest, Cerebral Performance Category (CPC), and in-hospital mortality.

Location was classified into three categories based on where OHCA occurred: public space, private space, and in the presence of emergency medical staff (EMS). The third category (EMS) was defined as the development of cardiac arrest on EMS arrival with spontaneous circulation on initial EMS evaluation, as many cases occur in ambulances.

### 2.4. Definitions and Outcome Measures

First, we divided the enrolled patients into four groups according to seasons (spring, summer, autumn, and winter) based on the definitions by the Japan Meteorological Agency. We defined spring as the period between 1 March and 31 May; summer as between 1 June and 31 September; autumn as between 1 September and 31 November; and winter as between 1 December and 30 February.

ROSC was defined as the return of spontaneous circulation that lasted for at least 1 min [8].

The primary outcomes were in-hospital mortality and favorable neurological outcomes at hospital discharge. A favorable neurological outcome was defined as a score of 1 or 2; an unfavorable neurological outcome was defined as a CPC score of 3, 4, or 5.

### 2.5. Statistical Analysis

For univariate analysis, we used Student’s t-test or the Mann–Whitney U test to compare continuous variables, and we used the χ2 test or Fisher’s exact test to compare categorical variables, as appropriate. We used a one-way analysis of variance to assess the differences in characteristics and outcomes among the four groups. We then evaluated the differences in the outcomes between the winter and non-winter seasons. Multivariable logistic regression analysis was performed to assess clinical and neurological outcomes. The variables incorporated into the model were age, sex, body temperature, witnessed status, bystander CPR status, shockable rhythm, estimated low-flow time, ROSC before hospital arrival, cardiac arrest location, cause of cardiac arrest, receiving percutaneous coronary intervention (PCI), and winter, which were based on the clinical perspective (subject-matter knowledge) and the number of outcomes (10 events per variable rule). The variables of bystander defibrillation and/or AED use were not included in the model because of multicollinearity. These variables were selected based on clinical judgment.

All statistical analyses were performed using the R software (version 4.2.1; R Foundation for Statistical Computing, Vienna, Austria) and a commander module incorporating frequently used biostatistical functions. Differences were considered statistically significant at two-tailed *p*-values < 0.05.

## 3. Results

**Figure 1:**
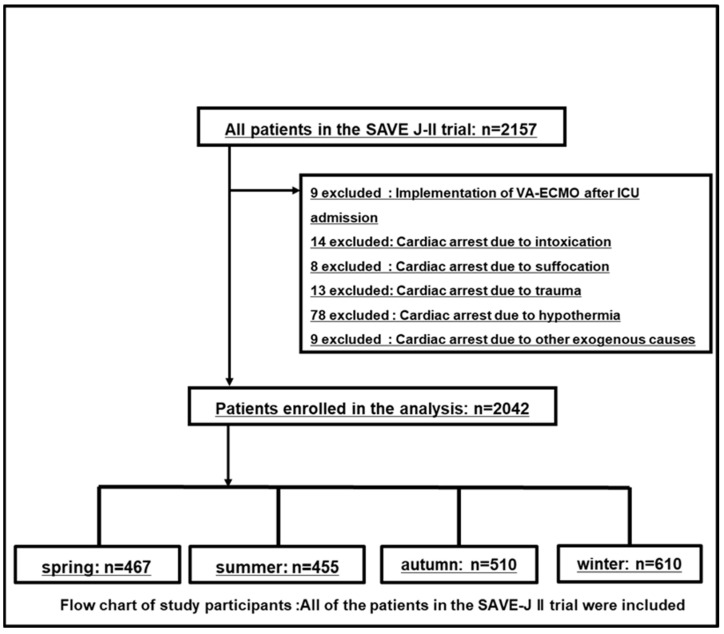
Flow chart of study participants. All patients in the Study of Advanced Life Support for Ventricular Fibrillation with Extracorporeal Circulation in Japan (SAVE-J) II trial were included. Nine patients were excluded because they received ECPR after ICU admission, fourteen patients were excluded because their cause of cardiac arrest was intoxication, eight patients were excluded because their cause of cardiac arrest was suffocation, thirteen patients were excluded because their cause of cardiac arrest was trauma, seventy-eight patients were excluded because their cause of cardiac arrest was accidental hypothermia, and three patients were excluded because their cause of cardiac arrest was other exogenous cause. VA-ECMO, venoarterial extracorporeal membrane oxygenation; ICU, intensive care unit.

Of the 2157 potentially eligible patients, 2042 patients with OHCA who underwent ECPR were included in the analysis (Figure 1). The study population was divided based on season: spring, 467 (22.8%); summer, 455 (22.3%); autumn, 510 (25.0%); and winter, 610 (29.9%). The baseline characteristics of the patients and outcomes according to the season are shown in Table 1. The mean patient age was 59 years, and 82.7% in all groups were males. Statistically significant differences among the four groups were observed regarding age, body temperature, and OHCA location. The body temperature was lowest in winter (spring 34.7 °C vs. summer 35.6 °C vs. autumn 35.0 °C (14.4%) vs. winter 34.3 °C (12.6%), *p* < 0.001). All remaining characteristics were similarly distributed. There was no significant difference in in-hospital mortality (spring 337 [73.6%] vs. summer 337 [72.2%] vs. autumn 340 [74.7%] vs. winter 456 [74.9%], *p* = 0.649), and in the rate of favorable neurologic outcome (spring 66 [14.3%] vs. summer 60 [13.4%] vs. autumn 73 [14.4%] vs. winter 76 [12.6%], *p* = 0.144).

Table 2 shows the results of the comparison of characteristics and the univariate analysis results of the outcomes between the winter and non-winter periods. The mean age, mean body temperature, and location showed the same trends as in the comparison of the four seasons, as shown in Table 1. In winter, the body temperature was lower than in the other seasons (35.0 °C [34.30, 36.00] vs. 34.3 °C [33.80, 35.80]; *p* < 0.001); and bystander CPR was performed more commonly in winter than in the other seasons (77.5% vs. 61.6%; *p* = 0.037). There were no significant differences in the outcomes between the two groups.

Table 3 shows the results of univariate and multivariate analyses of in-hospital mortality. Although body temperature and bystander CPR were significantly different in the univariate analysis, after controlling for age, sex, body temperature, witnessed status, bystander CPR status, shockable rhythm, estimated low-flow time, ROSC before hospital arrival, cardiac arrest location, cause of cardiac arrest, PCI, and winter, these factors were not significantly associated with in-hospital mortality. Thus, age (odds ratio [OR] 1.03; 95% confidence interval [CI], 1.02–1.03; *p* <0.001), sex (male) (OR 1.50; 95% CI, 1.06–2.11; *p* = 0.020), shockable rhythm (OR 0.62; 95% CI, 0.46–0.83; *p* = 0.002), cardiogenic (OR 0.53; 95% CI, 0.36–0.77; *p* = 0.001), low-flow time (OR 1.01; 95% CI, 1.00–1.01; *p* = 0.032), and PCI (OR 0.71; 95% CI, 0.54–0.95; *p* = 0.02) were significantly associated with in-hospital mortality. Winter as a seasonal factor was not significantly associated with worsened in-hospital mortality (OR 0.98; 95% CI, 0.74–1.29; *p* = 0.855).

Table 4 shows the results of the univariate and multivariate analyses of favorable neurological outcomes. Although body temperature showed a statistically significant difference in the univariate analysis, after controlling for age, sex, body temperature, witnessed status, bystander CPR status, shockable rhythm, estimated low-flow time, ROSC before hospital arrival, cardiac arrest location, cause of cardiac arrest, receiving PCI, and winter, it was not significantly associated with favorable neurological outcomes. Age (OR 0.97; 95% CI, 0.96–0.98; *p* < 0.001), sex (male) (OR 0.64; 95% CI, 0.44–0.97; *p* = 0.030), bystander CPR (OR 1.58; 95% CI, 1.09–2.32; *p* = 0.018), and initial shockable rhythm (OR 1.90; 95% CI, 1.23–2.99; *p* = 0.004) were significantly associated with favorable neurologic outcomes. Winter as a seasonal factor was not significantly associated with favorable neurologic outcomes (OR 0.95; 95% CI, 0.65–1.39; *p* = 0.807).

## 4. Discussion

In this retrospective multicenter observational study, we assessed seasonal differences in clinical and neurological outcomes among 2042 patients with OHCA treated with ECPR. Our findings indicate that, relative to non-winter cases, winter OHCA cases were higher, with lower body temperature, and there were more cases with bystander CPR. However, there were no seasonal differences in in-hospital mortality or neurological outcomes even after adjusting for prehospital confounding factors.

To our knowledge, this is the first study to investigate seasonal variations in patients with OHCA who underwent ECPR. Recognizing the number of patients requiring ECPR is crucial because ECPR requires significant human and healthcare resources. Therefore, analyzing the additional risk of seasonal differences would increase the potential to optimize ECPR implementation and indirectly improve the outcomes of patients receiving ECPR. However, it is difficult to conduct a well-designed randomized controlled trial to detect seasonal differences in patients undergoing ECPR. A retrospective study is suitable for this purpose.

In this study, patients in the winter group tended to have clinically unfavorable conditions (e.g., older age). However, there were no significant differences in the outcomes between the winter and non-winter seasons. Therefore, based on our findings, it can be inferred that physicians should not abandon the option of ECPR for patients with OHCA based solely on seasonal factors, even if it entails a slightly longer low-flow time. Although we cannot alter the risk of the season itself, adequate recognition of the risk and its characteristics enables us to control the intensity of ECPR. Furthermore, such an approach could serve as a foundation for further extensive research, including investigations on temperature-related factors. This study has the potential to initiate large-scale research in the future.

Cold weather has been associated with mortality in patients with cardiovascular and/or respiratory diseases [10,11], possibly due to the higher occurrence of OHCA in winter. Increased blood viscosity in cold conditions has been reported to lead to coronary artery thrombosis with high mortality [12]. Some reports have also suggested that OHCA prognosis is poorer at low ambient temperatures during winter than in other seasons [13,14,15,16,17,18,19,20]. In contrast with previous studies, no statistically significant differences in mortality and neurological outcomes were observed between the winter and non-winter seasons in this study. A possible explanation for this discrepancy is the influence of ambient temperature on patients with OHCA after ROSC. Lower body temperatures could lead to good neurological outcomes [21]. TTM is recommended for post-cardiac arrest syndrome [11], but in a previous study, TTM at a lower temperature was not beneficial for neurological outcomes and mortality [22]. However, this study only included OHCA patients who underwent ECPR. In patients treated with ECPR, body temperature could be strictly controlled using the heat exchanger in the extracorporeal membrane oxygenation circuit, and several studies have reported the association between improved outcomes in patients with OHCA and the combination of ECPR and TTM [15,23,24,25,26,27]. The body temperature of patients is thought to be colder in the winter season, as shown in this study (35.0 °C vs. 34.3 °C). This trend led to better outcomes in patients with OHCA in winter. However, there are daily and regional variations in temperature, and it is unclear whether these variations affect body temperature. Furthermore, the inclusion criterion was patients who received ECPR, and a comparative analysis of patients who did not undergo ECPR was not feasible. However, this study included only OHCA patients who underwent ECPR, and thus the severity of patients could be higher than in other previous studies, leading to selection bias. Another possible explanation is the cause of body temperature changes. Future studies should consider patients receiving and not receiving ECPR to evaluate the progression of body temperature changes from hospital arrival, categorized by season.

Considering the results of this study, physicians should not predict the outcomes of patients with OHCA who are ECPR candidates based solely on seasonal factors, even if it entails a slightly longer low-flow time. The severity of acute coronary syndrome and the specific proportion of complications that are more prevalent during winter, such as pneumonia, were not examined. To determine whether the prognosis of patients with OHCA worsens during winter, larger-scale studies accounting for factors such as temperature and incidence of complications are needed.

The strength of our study is that we directly assessed 2042 patients receiving ECPR using a large-scale multicenter database from the SAVE-J II study comprising 36 hospitals in Japan. Furthermore, the four distinct seasons in Japan provide an ideal setting for the seasonal assessment of disease prognosis. Many patients with OHCA who underwent ECPR were included, which enabled us to detect small differences between the seasonal groups.

However, this study had several limitations that should be considered when interpreting our findings. First, its retrospective design was prone to residual confounding factors and the risk of type I error and/or survivor bias. Furthermore, the decision to implement ECPR was not uniformly protocolized. The information was based on the Japanese seasonal conditions; therefore, these results cannot be adapted to other regions. Although there is a study with a similar division of seasons, this result cannot be generalized. Second, in this study, we divided the year into four seasons, but these seasons include various factors that affect the outcome of patients with OHCA, such as temperature, humidity, and hours of sunlight. It has been reported that the proportion of witnessed cardiac arrests is higher in summer than winter [15]. Although the total number of OHCA cases with witnesses was not significantly different in this study, there were slightly more cases with EMS present in winter than in other seasons. OHCA occurrence in the presence of people skilled in resuscitation may have resulted in good CPR before arrival at the hospital. The location of the cardiac arrest may be a factor in the implementation of ECPR, and in winter, patients with a better prognosis may have been selected for ECPR. The possibility of selection bias must also be considered. The relationship between the season and this factor is unclear, and more detailed studies are necessary.

We did not collect this information; therefore, it remains unclear whether prognostic factors should be considered. More detailed data collection (e.g., the peak temperature per day, differences) is needed to examine what winter-related factors contribute to patient outcomes. During the observation period of this study, there may have been regions experiencing abnormal weather conditions, such as mild winters or cool summers. Such factors may have potentially influenced our results. The impact of accidental lower ambient body temperature on neurological outcomes may differ from that of lower temperature caused by TTM. Third, we did not investigate treatment after hospitalization in this study. ECPR management varies among facilities, and the factors contributing to prognosis should be investigated. Fourth, several seasonal factors, such as sunlight hours and temperature, vary by region. Furthermore, there may be differences in population distribution and medical care, and the region where UHCA occurred might have influenced the prognosis. Although the season is not a variable that can be altered, based on the findings of our epidemiological study, further elucidation of the mechanisms that can result in improved patient survival outcomes is expected.

## 5. Conclusions

In this study, we evaluated seasonal differences in the outcomes of patients with OHCA treated with ECPR, but no seasonal differences were observed. Further large-scale studies are necessary to investigate the cause of this result and develop a therapeutic intervention.

## Figures and Tables

**Table 1:** (**A**) Baseline characteristics (comparing seasons); (**B**) outcome (comparing seasons).

**(A)**						
**Season**						
**Variables**	**Overall** **n = 2042**	**Spring** **n = 455**	**Summer** **n = 455**	**Autumn** **n = 510**	**Winter** **n = 610**	***p***
Age	59 (14)	59 (13)	58 (15)	59 (13)	61 (13)	0.002
Sex (Male),n (%)	1688 (82.7%)	383 (82.0%)	389 (85.5%)	417 (81.8%)	499 (81.8%)	0.350
Body temperature	34.8 (2.06)	34.7 (1.93)	35.6 (1.37)	35.0 (1.64)	34.3 (2.72)	<0.001
Witness, n (%)	1596 (78.5%)	362 (78.0%)	348 (76.5%)	396 (78.0%)	490 (80.7%)	0.388
Bystander CPR, n (%)	1164 (58.1%)	253 (55.1%)	252 (56.2%)	289 (58.1%)	370 (61.6%)	0.153
Location, n (%)						0.022
EMS	248 (12.1%)	46 (9.9%)	44 (9.7%)	61 (12.0%)	97 (15.9%)	
Private	803 (39.3%)	184 (39.4%)	185 (40.7%)	212 (41.6%)	222 (36.4%)	
Public	991 (48.5%)	237 (50.7%)	226 (49.7%)	237 (46.5%)	291 (47.7%)	
Initial rhythm, n (%)						0.311
Asystole	183 (9.1%)	45 (9.9%)	42 (9.3%)	35 (6.9%)	61 (10.1%)	
PEA	549 (27.3%)	116 (25.6%)	122 (27.0%)	145 (28.7%)	166 (27.5%)	
VF	1240 (61.6%)	284 (62.6%)	282 (62.4%)	317 (62.8%)	357 (59.2%)	
VT	42 (2.1%)	9 (2.0%)	6 (1.3%)	8 (1.6%)	19 (3.2%)	
AED, n (%)	1217 (60.2%)	284 (61.7%)	278 (61.6%)	305 (60.0%)	350 (58.2%)	0.611
Defibrillation, n (%)	1282 (63.7%)	293 (64.5%)	288 (63.7%)	325 (64.4%)	376 (62.4%)	0.873
Prehospital ROSC, n (%)	263 (13.1%)	71 (15.5%)	54 (12.1%)	59 (11.7%)	79 (13.3%)	0.294
PCI, n (%)	841 (41.2%)	188 (40.3%)	196 (43.1%)	202 (39.6%)	255 (41.8%)	0.692
Cardiogenic, n (%)	1552 (76.0%)	349 (74.7%)	342 (75.2%)	393 (77.1%)	468 (76.7%)	0.784
**(B)**						
**Season**						
**Variables**	**Overall** **n = 2042**	**Spring** **n = 455**	**Summer** **n = 455**	**Autumn** **n = 510**	**Winter** **n = 610**	***p***
Favorable neurological outcome, n (%)	275 (13.6%)	66 (14.3%)	60 (13.4%)	73 (14.4%)	76 (12.6%)	0.809
In-hospital mortality, n (%)	1502 (73.6%)	337 (72.2%)	340 (74.7%)	369 (72.4%)	456 (74.9%)	0.634

CPR, cardiopulmonary resuscitation; EMS, emergency medical service; AED, automated external defibrillator; PCI, percutaneous coronary intervention; ROSC, return of spontaneous circulation. Data are presented as mean (SD). Developing cardiac arrest after EMS arrival with the presence of spontaneous circulation on initial EMS evaluation.

**Table 2:** (**A**) Baseline characteristics (comparing winter with the other seasons); (**B**) outcome (comparing winter with the other seasons).

**(A)**			
**Season**			
**Variables**	**Other** **n = 1475**	**Winter** **n = 673**	***p***
Age	58(14)	61(13)	<0.001
Sex (Male), n (%)	1189 (83.0%)	499 (81.8%)	0.502
Body temperature	35.01(1.68)	34.30(2.72)	<0.001
Witness, n (%)	1106 (77.5%)	490 (80.7%)	0.106
Bystander CPR, n (%)	794 (56.6%)	370 (61.6%)	0.037
Location, n (%)			0.002
EMS	151 (10.5%)	97 (15.9%)	
Private	581 (40.6%)	222 (36.4%)	
Public	700 (48.9%)	291 (47.7%)	
Initial rhythm, n (%)			0.090
Asystole	122 (8.6%)	61 (10.1%)	
PEA	383 (27.1%)	166 (27.5%)	
VF	883 (62.6%)	357 (59.2%)	
VT	23 (1.6%)	19 (3.2%)	
AED, n (%)	867 (61.1%)	350 (58.2%)	0.229
Defibrillation, n (%)	906 (64.2%)	376 (62.4%)	0.428
Prehospital ROSC, n (%)	184 (13.0%)	79 (13.3%)	0.901
Low-flow time	56 (46, 69)	55 (45, 68)	0.302
PCI, n (%)	586 (40.9%)	255 (41.8%)	0.711
Cardiogenic, n (%)	1084 (75.7%)	468 (76.7%)	0.620
Favorable neurological outcome, n (%)	199 (14.0%)	76 (12.6%)	0.391
In-hospital mortality, n (%)	1046 (73.0%)	456 (74.9%)	0.390
**(B)**			
**Season**			
**Variables**	**Other** **n = 1475**	**Winter** **n = 673**	***p***
Favorable neurological outcome, n (%)	199 (14.0%)	76 (12.6%)	0.391
In-hospital mortality, n (%)	1046 (73.0%)	456 (74.9%)	0.390

CPR, cardiopulmonary resuscitation; EMS, emergency medical service; AED, automated external defibrillator; PCI, percutaneous coronary intervention; ROSC, return of spontaneous circulation, Data are presented as mean (SD). Developing cardiac arrest after EMS arrival with the presence of spontaneous circulation on initial EMS evaluation.

**Table 3:** Multivariate analysis for in-hospital mortality.

	In-Hospital Mortality		
Characteristic	Adjusted OR [95% CI]		*p*-Value
Age	1.02	[1.01, 1.03]	<0.001
Sex (Male)	1.50	[1.06, 2.11]	0.020
Body temperature	1.01	[0.935, 1.09]	0.741
Witness	0.85	[0.60, 1.18]	0.326
Bystander CPR	1.02	[0.77, 1.34]	0.896
EMS	0.70	[0.46, 1.10]	0.118
Shockable rhythm	0.56	[0.41, 0.77]	<0.001
Low-flow time	1.01	[1.00, 1.01]	0.032
PCI	0.72	[0.54, 0.95]	0.020
Cardiogenic	0.53	[0.36, 0.77]	0.001
Winter	1.10	[0.82, 1.46]	0.530

CPR, cardiopulmonary resuscitation; OR, odds ratio; CI, confidence interval; PCI, percutaneous coronary intervention; EMS, emergency medical service; Developing cardiac arrest after EMS arrival with the presence of spontaneous circulation on initial EMS evaluation.

**Table 4:** Multivariate analysis for favorable neurologic outcomes.

	Favorable Neurologic Outcomes		
Characteristic	Adjusted OR [95% CI]		*p*-Value
Age	0.97	[0.96, 0.99]	<0.001
Sex (Male)	0.58	[0.38, 0.90]	0.014
Body temperature	0.97	[0.88, 1.08]	0.549
Witness	1.16	[0.74, 1.87]	0.537
Bystander CPR	1.58	[1.09, 2.32]	0.018
EMS	1.69	[0.97, 2.87]	0.056
Shockable rhythm	2.09	[1.36, 3.28]	<0.001
Low-flow time	1.34	[0.92, 1.97]	0.126
CAG	1.65	[0.99, 2.84]	0.063
Cardiogenic	0.90	[0.61, 1.31]	0.588
Winter	1.00	[0.95, 1.01]	0.571

CPR, cardiopulmonary resuscitation; OR, odds ratio; CI, confidence interval; CAG, coronary angiography; EMS, emergency medical service. Developing cardiac arrest after EMS arrival with the presence of spontaneous circulation on initial EMS evaluation.

## Data Availability

Third-party data restrictions apply to the availability of these data. Data were obtained from Wataru Takayama and are available from the authors with the permission of Wataru Takayama.
